# Status Epilepticus as a Consequence of Hemophagocytic Lymphohistiocytosis in a Previously Healthy Infant

**DOI:** 10.7759/cureus.6824

**Published:** 2020-01-30

**Authors:** Saeed M Nassar, Muath A AlBattah, Ahmad A Bukhari, Aryaf Alslimah, Assalh A Nahass

**Affiliations:** 1 Pediatric Intensive Care Unit, King Khaled University Hospital, Riyadh, SAU; 2 Pediatric Intensive Care Unit, King Khalid University Hospital, Riyadh, SAU; 3 Pediatric Intensive Care Unit, King Saud University, Riyadh, SAU

**Keywords:** pediatric intensive care unit, seizures, status epilepticus, genetics, pediatric critical care medicine, general pediatrics, hematology, neurology

## Abstract

Hemophagocytic lymphohistiocytosis (HLH) is a rare and fatal entity with an incidence rate of 1.2 cases per million people per year. HLH is explained as a highly destructive inflammatory consequence of rampant hypercytokinemia due to excessive lymphocyte-mediated activation of macrophages and histiocytes. Primary HLH is a product of genetic dysfunction and could be familial (five subtypes), syndromic immunodeficiency, or as a consequence of mutations predisposing a person to Epstein Barr Virus (EBV) infection. With secondary HLH, there is an identifiable cause provoking the inflammatory reaction, whether it is an infection, an autoimmune disease, or malignancy (particularly hematological). As a result of widespread cytokine deposition, systemic manifestations are seen with a variety of manifestations that can vary between cases.

This is a case of a patient who initially presented to the emergency department with fever, altered mentation, and gastroenteritis. Initial investigations showed non-anion gap metabolic acidosis, high white cell count, and deteriorating renal function. Further laboratory tests, bone marrow biopsies, and neurological imaging were conducted throughout the course of admission as the patient further deteriorated systemically. However, it's important to note the abundant neurological manifestations with a worsening level of consciousness and seizures, some of which were categorized as status epilepticus.

## Introduction

Hemophagocytic lymphohistiocytosis (HLH) is a fatal syndrome due to an unconstrained activation of lymphocytes (cytotoxic T cells and NK cells) and macrophages, spurring a hyperinflammatory response with a clinical manifestation of multiorgan dysfunction as a consequence of hypercytokinemia. This disease typically affects infants 18 months or younger, however, it may also be observed in adults and children. It has been characteristically divided into two subtypes, primary HLH and secondary HLH. Primary HLH (also regarded as familial HLH) is mostly autosomal recessive, although it could be sporadic, with respect to identified genetic mutations that halt the functions of proteins vital for the function of T cells and NK cells. Secondary HLH has no established genetic cause and thus has been attributed to immune-suppressing malignancies, infections, autoimmune diseases, as well as immunosuppressive agents [1-2]. HLH is a rare entity with an incidence rate of 1.2 cases per million people per year [3].

## Case presentation

A 10-month-old girl, with no past history of medical or surgical illness, presented to the emergency department (ED) with fever, altered mentation, vomiting, and loose stools. The fever was continuous and had a duration of one week, measured by the tympanic route, with a maximum temperature of 38.5 °C responding to antipyretics. It was associated with minimal symptoms of coryza, which resolved over the course of the fever. There were no associated rashes, night sweats, or weight loss according to the parents. She was assessed four days prior to her ED visit at a private clinic; they prescribed azithromycin for one week but the fever showed no signs of remission. A day later, three days prior to presentation, she started vomiting after each feed. She vomited at a frequency of six times per day, and it primarily consisted of food content. She also had loose stools occurring three times per day; it had mucus but no blood was observed. She had no associated abdominal pain or jaundice at the time of presentation. A day prior to her ED visit, her parents noted a change in her level of consciousness associated with decreased feeding and activity. There were no perceived motor or sensory impairments and no other neurological deficits. She is the product of a full-term uneventful pregnancy and was thriving well on bottle feeding and diet for age. The patient was vaccinated up to her age per the national vaccination schedule.

During her initial physical evaluation, the patient was febrile at 38.9 ° C, with an elevated heart rate of 190 per minute and an elevated respiratory rate of 42 per minute. Upon inspection, she looked lethargic, lying on the bed with minimal activity, and she exhibited a Kussmaul breathing pattern. There were signs of moderate dehydration, such as pallor, mottled skin, and depressed fontanelle; otherwise, the physical examination was unremarkable. Initial investigations conducted at the ED have been added in Table 1. A chest X-ray was done as part of the septic screen and computed tomography (CT) brain was ordered to rule out meningitis, both of which were deemed unremarkable. Blood, cerebrospinal fluid (CSF), and urine cultures were ordered as part of the septic workup, all of which were negative. A provisional diagnosis of gastroenteritis with non-anion gap metabolic acidosis was made and the patient was started on fluids and antibiotics. She was kept on 190 ml/kg/day of fluids, including her maintenance, deficit, and ongoing losses. Sodium bicarbonate was also given for her metabolic acidosis. As for antibiotics, she was started on a broad spectrum regimen of ceftriaxone, vancomycin, and acyclovir. As cultures returned with a negative result, vancomycin and acyclovir were discontinued.

**Table 1:** Initial investigations done at the emergency department AST: Aspartate Aminotransferase; ALT: Alanine Transaminase; ALP: Alkaline Phosphatase; GGTP: Gamma-Glutamyltransferase; LDH: Lactate Dehydrogenase; LDH: Lactate Dehydrogenase; PCO2: Partial Pressure of Carbon Dioxide

Initial investigation	Value	Reference range
Hemoglobin	124 gm/L	95.0-135.0 gm/L
Hematocrit	38.7%	42-52%
Reticulocyte count	1.2%	0.5-2.5%
Leukocyte count	22.900 x10^9^/L	(6,000-18,000) x10^9^/L
Platelet count	434x10^9^/L	(140,00-450,0) x10^9^/L
LDH	>1000 unit/L	84-246 unit/L
Serum creatinine	79 µmol/L	18-35 µmol/L
Serum blood urea nitrogen	7.3 mmol/L	1.4-6.8 mmol/dl
pH	7.14	7.3-7.4
PCO2	27.7 mmHg	41-54 mmHg
Serum bicarbonate	11 mEq/L	22-29 mEq/L
Serum sodium	142 mmol/L	135-145 mmol/L
Serum potassium	5.3 mmol/L	4.1-5.3 mmol/L
Serum glucose	26.2 mmol/L	2.8-4.4 mmol/L
Serum lactic acid	11.8 mmol/L	0.5-2.2 mmol/L
Serum osmolality	318 mOsm/L	285-295 mOsm/L
AST	>1000 unit/L	10-31 unit/L
ALT	>1000 unit/L	20-65 unit/L
ALP	346 unit/L	134-513 unit/L
GGT	110 U/L	5-55 unit/L
Total bilirubin	2.78 µmol/L	3.00-17.00 µmol/L
Direct bilirubin	1.25 µmol/L	0.00-5.00 µmol/L
Indirect bilirubin	2 µmol/L	2-17 µmol/L

**Figure 1:**
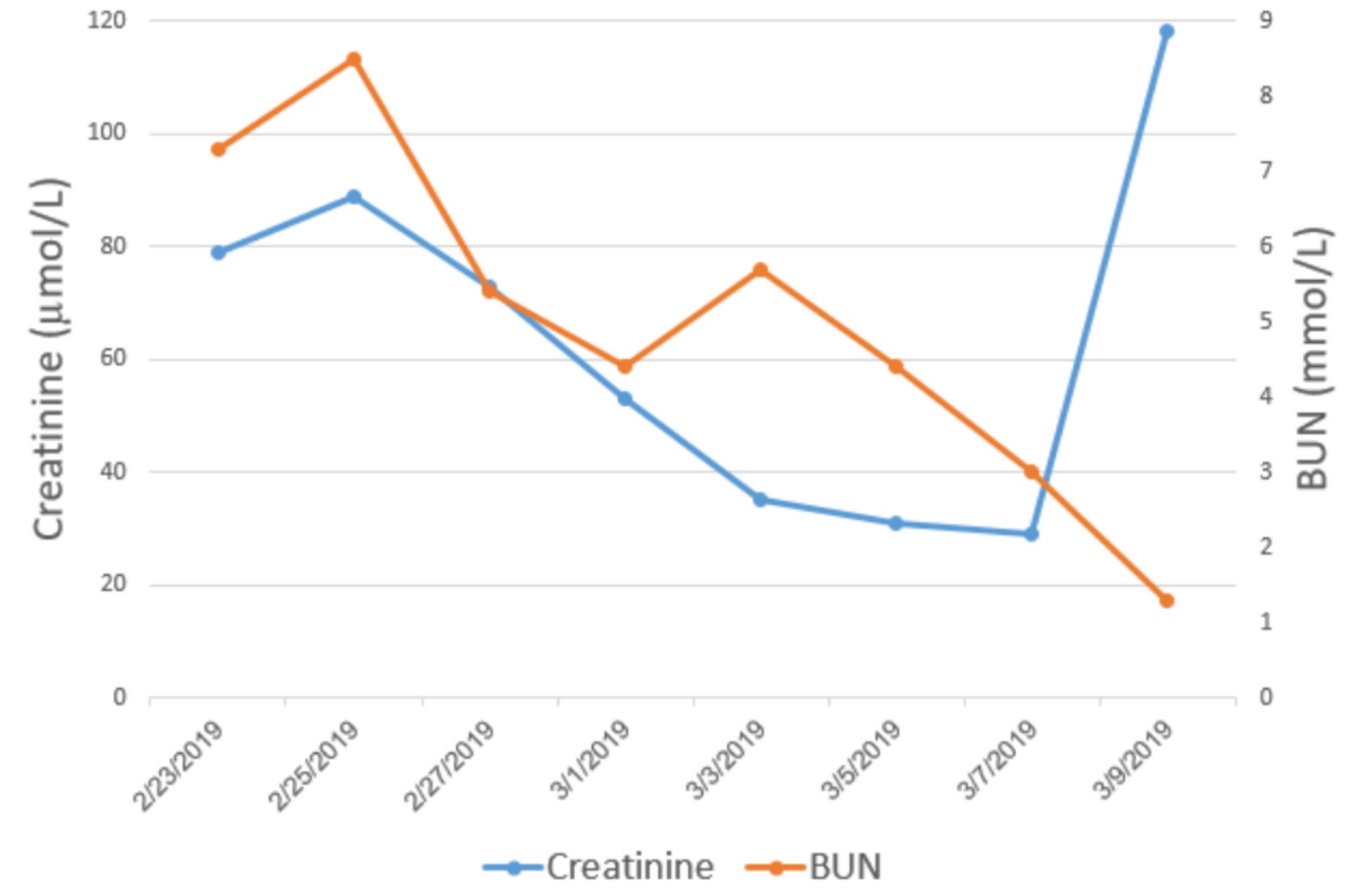
Trend of renal function throughout the hospital course of the patient BUN: blood urea nitrogen

**Figure 2:**
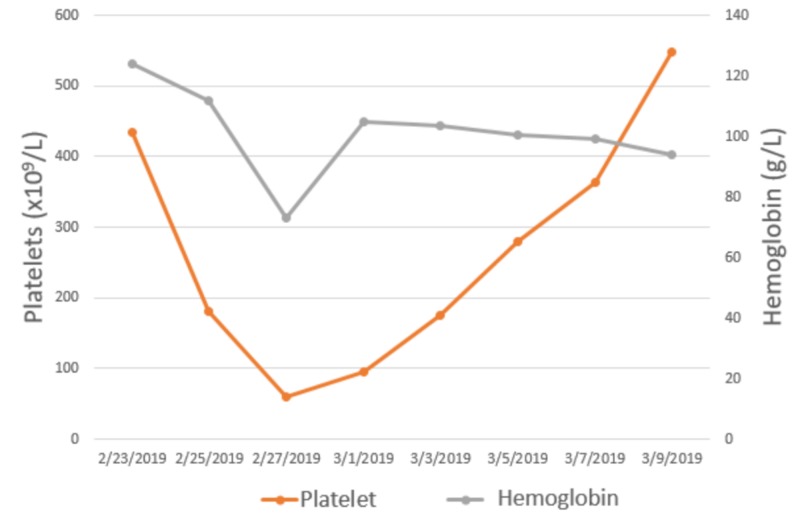
Trend of hemoglobin and platelet levels throughout the hospital course of the patient

**Figure 3:**
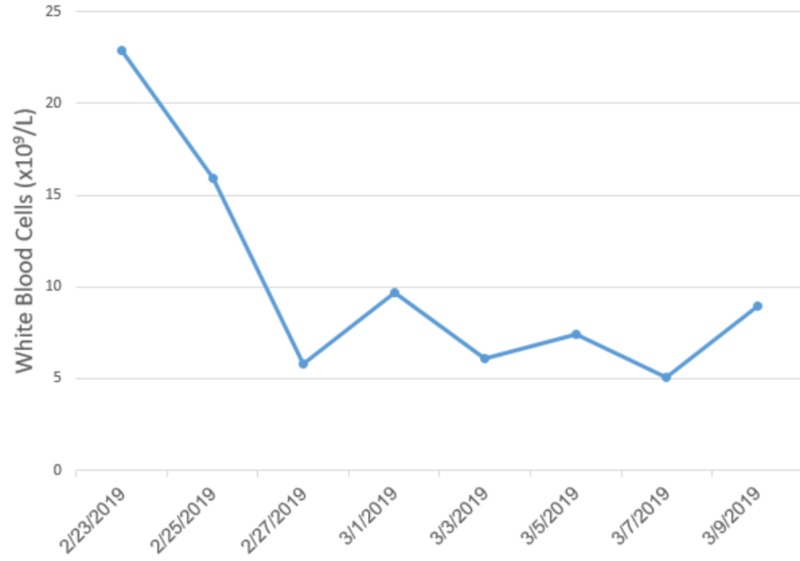
Trend of white blood cell counts throughout the hospital course of the patient

She was immediately transferred to the pediatric intensive care unit (PICU) due to her unstable clinical impression. Over the course of her admission, she developed acute kidney injury (AKI), bicytopenia (constituted of hemoglobin and platelet deficiency), acute liver failure, and disseminated intravascular coagulation (DIC). She was found to have AKI at the day of presentation with creatinine levels of 79 µmol/l and a blood urea nitrogen (BUN) 7.3 mmol/l (Figure 1), which could be attributed to the severe dehydration that the patient developed, a confounding factor initially as to whether or not HLH was involved. She then progressed to develop bicytopenia with thrombocytopenia and anemia. Her platelet count was 92x109/L, which steadily declined to 65x109/L by the fifth day of admission (Figure 2). She became anemic by the third day of admission where her Hgb dropped to 73 g/l. White blood cell counts were initially high; eventually, they declined but not to the point of leukopenia (Figure 3).

**Figure 4:**
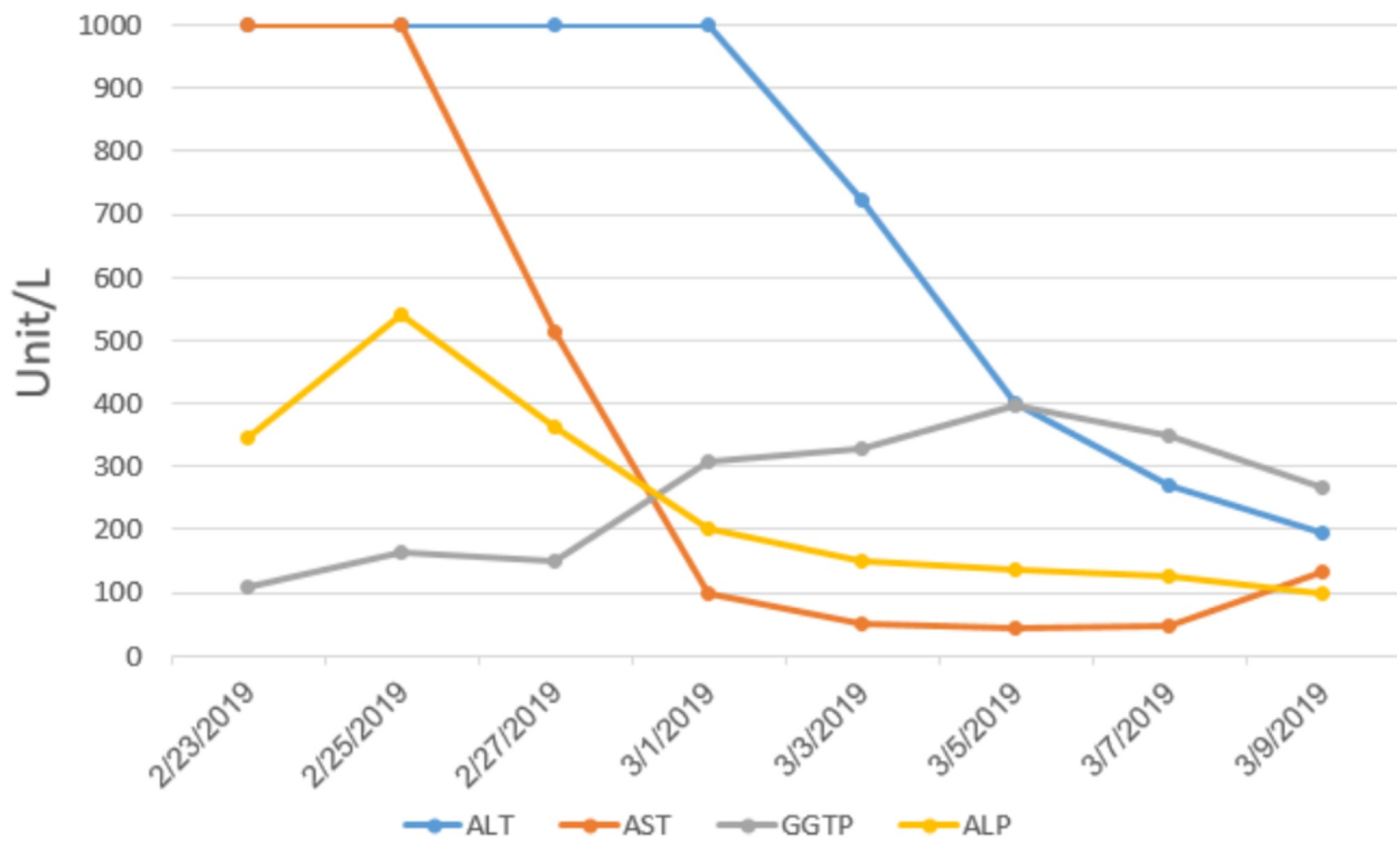
Trend of liver enzymes throughout the hospital course of the patient ALT: Alanine Transaminase; AST: Aspartate Aminotransferase; GGTP: Gamma-Glutamyltransferase; ALP: Alkaline Phosphatase

**Figure 5:**
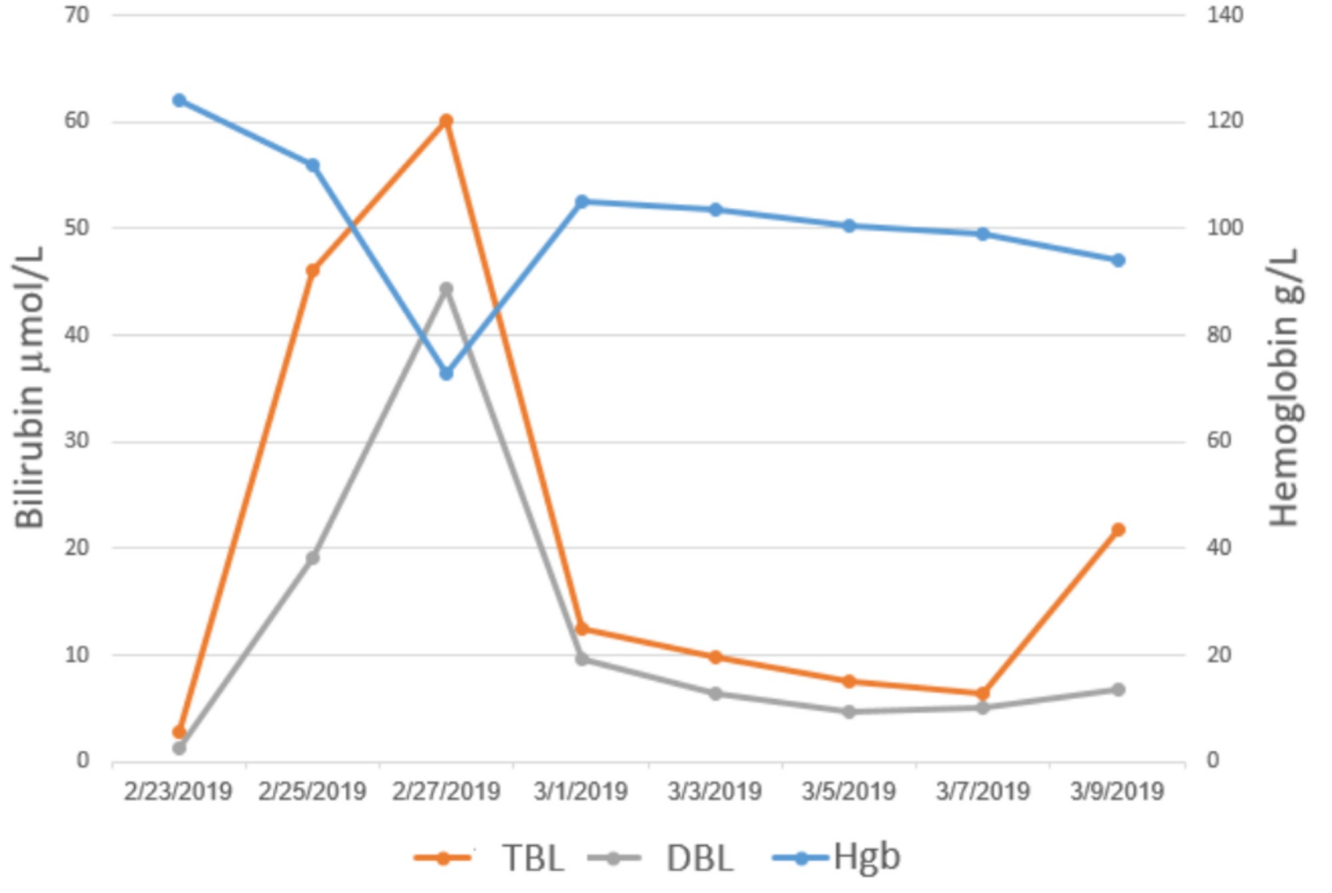
Trend of bilirubin and hemoglobin levels throughout the hospital course of the patient Hgb: Hemoglobin; TBL: Total Bilirubin Level; DBL: Direct Bilirubin Level

She also developed acute liver failure during her second day of admission, with alanine transaminase (ALT) and aspartate aminotransferase (AST) greater than 1000 u/l, respectively, gamma-glutamyltransferase (GGTP) 164 u/l and ALP 542 u/l (Figure 4). She also had altered synthetic liver markers with hyperbilirubinemia (total of 46.02 umol/l, direct at 19.17 umol/l, and indirect 27 mcmol/l) (Figure 5), hypoalbuminemia (22.34 g/l), and hypertriglyceridemia (1.68 mmol/l). Her deleterious liver functions, in concordance with her primary ailment, showed labs suggestive of disseminated intravascular coagulation (DIC) due to hypofibrinogenemia (1.51 g/l) and elevated international normalized ratio (INR) of 1.67s, prothrombin time of 42.6s, activated partial thromboplastin time of 54.4s, and D dimers at 1.3 mcg/ml. She was promptly managed with fresh frozen plasma and intramuscular vitamin K. Other tests included lactate dehydrogenase, which was over 1000, and ferritin, which was severely elevated at 8,413.5 mcg/l.

**Figure 6:**
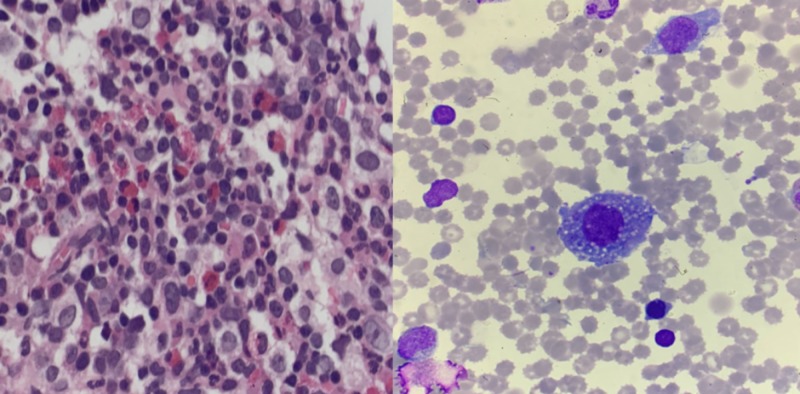
This cellular bone marrow shows active trilineage hematopoiesis with frequent histiocytes and significant hemophagocytic activities.

The leading differential according to the clinical and laboratory evidence was mainly toward hemolytic uremic syndrome, rather than thrombotic thrombocytopenic purpura due to her afebrile state, which was ruled out by the normal reticulocyte count and lack of schistocytes on the peripheral blood smear. To better understand and differentiate the primary cause of her bone marrow suppression: lymphocyte subset analysis, bone marrow aspiration (BMA), and polymerase chain reaction for a variety of viruses (with a focus on cytomegalovirus (CMV) and EBV) were conducted. The results were significant, showing signs of HLH. Cluster of differentiation 4 (CD4) and natural killer cell activity were significantly reduced. The EBV test results were reactive for immunoglobulin G (IgG) viral capsid antigen and IgG Epstein Barr nuclear antibody; other viral antigens/antiviral antibodies were non-reactive. Autoimmune markers, such as antinuclear antibodies and complement levels, were unremarkable. The BMA was positive for hemophagocytic activity and frequent histiocytes (Figure 6), hence, a diagnosis of HLH was reached secondary to EBV infection. She was promptly commenced on an intravenous immunoglobulin (IVIG) 0.5 g/kg and dexamethasone regimen to reduce the inflammatory burden while genetic tests were pending.

**Figure 7:**
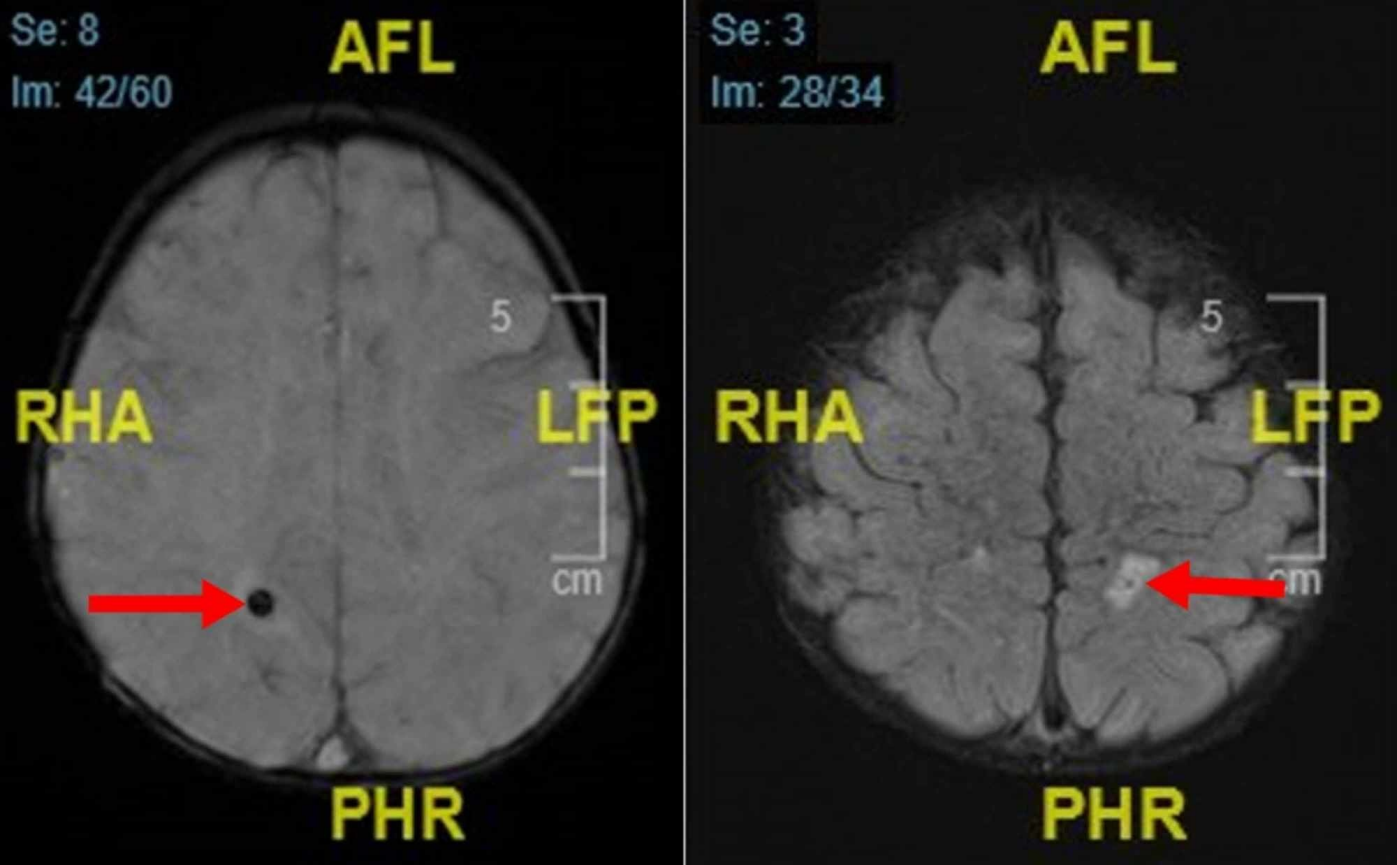
Bilateral signal alteration involving the subcortical and deep white matter seen in the background of mild cerebral atrophy. Isolated focal signal alteration of the splenium of the corpus callosum.

During her clinical course, she was intubated due to a further deterioration of her level of consciousness, represented by a drop of her Glasgow Coma Scale (GCS) from 13 to 8, leaving her unable to maintain her airway. This prompted an immediate CT and MRI (Figure 7), and it was followed by a reassessment of her cerebrospinal fluid (CSF); pathology reports showed few histiocytes. She was extubated a week later due to an interval improvement of her clinical status and transferred to the ward. On the 12th day of admission, she developed atonic seizures, twice within eight hours, associated with cyanosis, and the duration deemed it to be status epilepticus where she was managed with levetiracetam 20 mg/kg/day, with maintenance 10 mg/kg/day, and transferred back to the PICU. On the 13th day of admission, she developed an episode of status epilepticus with tonic-clonic seizures cyanosis and bradycardia for which the pediatric advanced life support (PALS) protocol was initiated, however, she developed asystole and expired despite resuscitative efforts (post-convulsive cardiopulmonary arrest). Whole-exome sequencing was sent out prior to her passing to rule out primary HLH, which came back negative a month later, ruling out primary HLH.

## Discussion

HLH is a highly destructive inflammatory consequence of rampant hypercytokinemia due to excessive lymphocyte-mediated activation of macrophages and histiocytes. It is divided into two distinct categories based on the pathophysiology of the said inflammatory response, primary and secondary. Primary HLH is a product of genetic dysfunction and could be familial (five subtypes), syndromic immunodeficiency, or a consequence of mutations predisposing a person to EBV infection. With secondary HLH, there is an identifiable cause provoking the inflammatory reaction, be it an infection, autoimmune disease, or malignancy (particularly hematological) [2]. Henceforth, the diagnosis of HLH can be made based on genetic testing, clinical manifestations, laboratory values, and bone marrow aspiration.

The most commonly recognized cause of secondary HLH as per the literature is an infection, predominantly by viruses. EBV, a deoxyribonucleic acid (DNA) virus originating from the genus Herpesviridae, is the most implicated organism. HLH secondary to EBV typically presents as a fulminant acute infection with excessive activation of CD8 cytotoxic T cells, contrary to other variants of EBV complications that result from the activation by B cells (such as infectious mononucleosis) [4]. This fatal phenomenon forebodes a mortality rate of 30%-50%, hence, immediate diagnosis and therapy is integral [5]. The HLH-2004 guidelines mandate that five of eight clinical and laboratory criteria must be met to rule out differentials. The criteria include fever, splenomegaly, pan/bicytopenia, hypertriglyceridemia (or hypofibrinogenemia or both), hemophagocytosis (seen in bone marrow, spleen, or lymph nodes without evidence of malignancy), low natural killer cell activity, hyperferritinemia, and increased levels of soluble CD25 (that is, soluble interleukin-2 receptor) [6]. Prognosis is hindered by young age and neurological manifestations. Survival rates in those under six months are at 41% whereas those over six months have a 65% survival rate. Neurological manifestations with HLH drop the survival rate to 40% [6].

It’s important to keep a wide differential, taking into consideration every clinical and laboratory test, as HLH can be very perplexing, with an atypical presentation, or when there are confounding factors leading the differential to more common diseases/entities. Our patient presented with severe gastroenteritis and signs of severe dehydration due to an acute EBV infection with no other physical signs of EBV or HLH. A similar case was observed by Fujiwara et al., where a patient presented with EBV-induced hemorrhagic gastroenteritis with no pharyngitis, lymphadenopathy, splenomegaly, or hepatic dysfunction [7].

Central nervous system (CNS) manifestations with HLH are vague and variable; it’s been reported in 30%-73% of patients diagnosed with HLH as part of its natural history. The pathophysiology and clinical findings are still being sought after. However, there’s a consensus that CNS involvement is defined by the presence of anomalies on three grounds: clinical, biochemical (CSF), and radiological [8]. Pathologically it’s been divided into three stages. In the first stage, and the mildest form of the disease, there’s an infiltration of lymphocytes coupled with macrophages, which can also be observed in the CSF. In the second stage, there is a perivascular infiltration. In its third stage, diffuse infiltration of the brain tissue, as well as tissue necrosis, occurs [9]. In a study conducted by Horne et al., 193 patients with the neurological manifestations of HLH were retrospectively evaluated for their biochemical and physical implications. Fifty-two percent of patients showed abnormalities in their CSF with regards to elevated protein and/or cell counts, with cell counts being the commonest anomaly overall. As for clinical symptoms, the commonest were seizures, meningism, irritability, and altered level of consciousness. Seldom do we find focal neurological signs. Although CSF findings are important, only 70%-80% of these patients had abnormal CSF findings and almost a quarter of patients with clinical CNS findings have no alterations in their CSF. The majority of patients showed abnormalities on CT and MR imaging, gravitating around cerebral atrophy and white matter lesions with demyelination [10].

Our patient presented with abundant neurological manifestation with an altered level of consciousness at the time of presentation, progressing to multiple seizures over the course of admission, some of which progressed to status epilepticus. Status epilepticus is a continuous or recurrent seizure, whereas, if recurrent, no recovery is observed. The seizure must span a duration of more than or equal to five minutes based on clinical and/or electrophysiological activity [11]. Gupta et al. saw a case of HLH with refractory status epilepticus due to HIV, it is difficult to differentiate whether or not it could be due to human immunodeficiency virus (HIV) encephalopathy based on clinical grounds; nonetheless, it is important to keep a wide differential, as HLH requires a high index of suspicion [12].

The management of HLH is primarily a combination of steroids (dexamethasone in particular) and etoposide, along with managing the underlying cause if it is secondary. In primary HLH is important that a patient receives stem cell transplantation following resolution to enhance survival rate [4]. However, when children present with HLH involving the CNS, it is pertinent to begin intrathecal corticosteroids and methotrexate, as this has become the standard of care. The use of systemic therapy (steroids with etoposide) along with intrathecal is likely to yield the same result as with intrathecal therapy alone [13]. Many studies believe that the addition of hematopoietic stem cell transplantation (HSCT) could further prevent the long-term sequelae of CNS involvement [13-14]. Immediate management is essential, as it can help reduce the long-term complications of HLH on the neurological system of a developing child. Nineteen percent of patients with CNS involvement were reported to have epilepsy, learning difficulties, speech delay, attention deficit hyperactivity disorder (ADHD), or cranial nerve palsies [15].

## Conclusions

To conclude, HLH is a serious inflammatory disease that may lead to serious neurological manifestations. With the atypical presentation of HLH early in its onset, one must have a very high index of suspicion when it begins to reveal itself, especially when secondary causes make themselves clear with thorough investigations. Immediate management is essential to prevent long-term physical and neurological complications that may burden the quality of life of the patient or even lead to mortality.
